# Videoconference Fatigue: A Conceptual Analysis

**DOI:** 10.3390/ijerph19042061

**Published:** 2022-02-12

**Authors:** Nicola Döring, Katrien De Moor, Markus Fiedler, Katrin Schoenenberg, Alexander Raake

**Affiliations:** 1 Institute of Media and Communication Science, Technische Universität Ilmenau, 98693 Ilmenau, Germany; 2 Department of Information Security and Communication Technology, Norwegian University of Science and Technology, 7491 Trondheim, Norway; katrien.demoor@ntnu.no; 3 Department of Technology and Aesthetics, Blekinge Institute of Technology, 374 35 Karlshamn, Sweden; markus.fiedler@bth.se; 4 Department for Clinical Psychology and Psychotherapy, Bergische Universität Wuppertal, 42119 Wuppertal, Germany; schoenenberg@uni-wuppertal.de; 5 Institute for Media Technology, Technische Universität Ilmenau, 98693 Ilmenau, Germany; alexander.raake@tu-ilmenau.de

**Keywords:** video call, Zoom, Skype, Cisco Webex, Microsoft Teams, Big Blue Button, exhaustion, COVID-19 pandemic, computer-mediated communication, face-to-face-communication

## Abstract

Videoconferencing (VC) is a type of online meeting that allows two or more participants from different locations to engage in live multi-directional audio-visual communication and collaboration (e.g., via screen sharing). The COVID-19 pandemic has induced a boom in both private and professional videoconferencing in the early 2020s that elicited controversial public and academic debates about its pros and cons. One main concern has been the phenomenon of videoconference fatigue. The aim of this conceptual review article is to contribute to the conceptual clarification of VC fatigue. We use the popular and succinct label “Zoom fatigue” interchangeably with the more generic label “videoconference fatigue” and define it as the experience of fatigue during and/or after a videoconference, regardless of the specific VC system used. We followed a structured eight-phase process of conceptual analysis that led to a conceptual model of VC fatigue with four key causal dimensions: (1) personal factors, (2) organizational factors, (3) technological factors, and (4) environmental factors. We present this 4D model describing the respective dimensions with their sub-dimensions based on theories, available evidence, and media coverage. The 4D-model is meant to help researchers advance empirical research on videoconference fatigue.

## 1. Introduction

Videoconferencing (VC) is a type of online meeting that allows two or more participants from different locations to engage in live multi-directional audio-visual communication [1,2]. Several VC systems are available today that work with wired or wireless internet access on computers and mobile devices alike (e.g., BigBlueButton, BlueJeans Meetings, GoToMeeting, Microsoft Teams, Cisco Webex, Skype, and last, but not least the currently most popular system Zoom). VC is typically used to communicate and/or collaborate within and between organizations in work, business and educational contexts, but private uses with family and friends are also common.

### 1.1. Videoconference Fatigue

The COVID-19 pandemic-induced boom in videoconferencing in the early 2020s has boosted public debates. Despite advantages of videoconferencing [3], people expressed being overwhelmed, frustrated or exhausted by constant VC usage, which coined the term(s) “videoconference fatigue”, or “Zoom fatigue”. Hundreds of newspaper articles have been appearing world-wide that affirm the public that “Zoom fatigue is real” and provide tips on how to avoid it (e.g., to plan enough breaks in between VC). Renowned legacy media outlets such as the BBC, the Harvard Business Review, and the Wall Street Journal have covered Zoom fatigue [4,5,6]. Another indicator of the relevance of the phenomenon is its online representation: “Videoconference fatigue” has collected more than 18,000 and “Zoom fatigue” more than 600,000 hits on Google (as of November 2021). 

At the same time, researchers have started to re-investigate VC, particularly with an eye on the pandemic situation and so-called Zoom fatigue. Researchers from different fields such as psychology, communication, medicine, neuroscience, and technology have already published comments on the relevance and implications of Zoom fatigue in academic journals [7,8,9,10,11,12,13]. Additionally, the very first theoretical, methodological and empirical full papers are available: The first theoretical paper by Bailenson [14] elaborated five distinct nonverbal mechanisms as causal factors of Zoom fatigue: (1) mirror anxiety trigged by the self-view window on the screen, (2) sense of being physically trapped by the need to stay relatively immobile in the small field of view of the camera, (3) hypergaze as the experience of having all conference participants’ eyes staring at oneself during the whole meeting, (4) cognitive load related to actively producing readable nonverbal cues in front of the camera, and (5) cognitive load related to interpreting nonverbal cues of other meeting participants in their respective windows. One of the very first empirical papers, an online-survey by Fauville et al. [15] found correlations between these five mechanisms and a newly developed “Zoom Fatigue scale” [16]. Our analysis will show, however, that more and different factors might also play a significant role in causing Zoom fatigue.

### 1.2. Aim of This Conceptual Analysis Article

While it is natural and helpful that the extensive use of VC in times of the COVID-19 pandemic has triggered public debates and increased research activities on seemingly prevalent problems such as Zoom fatigue, it is also important to clarify and scrutinize the concept, its components and causes. Gaining better insight into possible causes of videoconference fatigue will help to establish healthy videoconference use. History has taught us that new and emerging information and communication technologies often create both exaggerated hopes as well as irrational fears, and not seldom come with misattributions, myths, and biased forecasts on their impact [17,18,19]. A broad and thorough review of existing evidence on causal factors is therefore needed to guide rational, healthy and best practices-based use of VC. For example, given the major impact of the pandemic on everyday life, it is not surprising that many people feel overwhelmed, frustrated and exhausted. What part of this experience is “videoconference fatigue” or rather “working from home fatigue” or simply “pandemic fatigue”? Furthermore, what exactly are the causes of videoconference fatigue? To what degree are they technology-related (e.g., related to the disruptions of nonverbal communication through cameras and screens described above) or purely of organizational nature (e.g., scheduling too many meetings, or not leaving enough breaks in-between them)? 

The aim of this conceptual analysis article is to contribute to the conceptual clarification of videoconference fatigue by focusing on assumed causes. We use the popular and succinct label “Zoom fatigue” interchangeably with the more generic label “videoconference fatigue” and define it as the experience of physical, cognitive and emotional fatigue and exhaustion related to videoconference use regardless of the specific VC system used [10,15]. While there is not much controversy around the experience of fatigue, causal factors are highly disputed and, hence, the focus of this conceptual analysis. Based on multiple academic and media sources, established theories and evidence from different disciplines we try to answer one main research question: *Which factors cause videoconference fatigue*? 

## 2. Materials and Methods

This conceptual analysis article explores the concept of videoconference fatigue. A “concept” is understood as an abstraction of a phenomenon that is defined by its components and their interrelations [20,21]. To identify and structure the main components and sub-components of video conference fatigue, a conceptual analysis was conducted along the phases reported in Table 1. This eight-phase model is a revised version of the approach by Bröder et al. [22]. This approach draws on elements from the approaches introduced by Rodgers [21] (pp. 77–102) and Jabareen [20]. While it shares, e.g., its emphasis on flexibility and its iterative character with the latter, it also differs from it in several ways (e.g., less structured approach for the deconstructing and categorizing of the concept, predominant emphasis on the literature in phase 1 instead of also interviewing external experts). Further, the adopted approach differs from a traditional structured or systematic review in several ways, for instance in terms of the search strategy, involvement of external experts as part of the iterative model refinement, and overall purpose.

In Phase 1, videoconference fatigue was selected as the target concept with a focus on causal factors (a review of Zoom fatigue definitions does already exist [23]). Both media articles and academic papers were regarded as relevant data sources and identified via systematic online searches. Key search terms (videoconference fatigue, Zoom fatigue, videoconference stress, videoconference exhaustion, videoconference burnout) were used with both the Google search engine and the Google Scholar database. The identified media articles and academic papers investigated in the conceptual analysis are referenced in the results section. In addition, we looked into previous review and conceptual analysis papers that explored the concept of fatigue in general [23] as well as papers that analyzed specific types of fatigue such as decision fatigue [24], driver fatigue [25] and museum fatigue [26]. 

**Figure 1:**
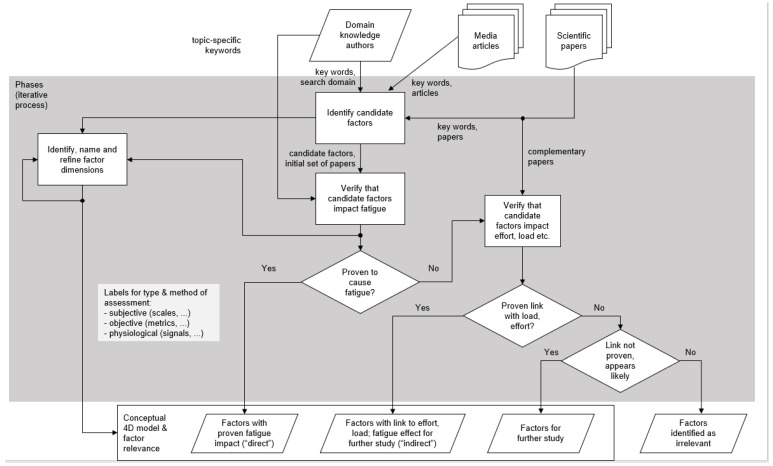
Flowchart of phases 2 to 6 of the conceptual analysis based on literature review and critical discussion within the research team.

In Phase 2, all authors read the identified sources carefully and contributed ideas on their categorization. The different suggestions for categorization were included in a 4D-model of videoconference fatigue in Phase 3. While the four dimensions of the new 4D model are generic and reflect typical conceptualizations of different fatigue phenomena such as driver fatigue [25] and museum fatigue [26,27], their sub-dimensions are videoconference-specific. In Phase 4, all sub-dimensions were carefully elaborated regarding their characteristics based on the sources mentioned above. Relevant theories, empirical evidence and media coverage are presented in *evidence tables* for all four dimensions (see Table 2, Table 3, Table 4 and Table 5). The selection of applicable theories was discussed among author and with external experts in phase 7 multiple times. We differentiate between *direct evidence* in the sense of data explicitly covering VC fatigue and *indirect evidence* in the sense of data from related fields. We are referring to media coverage and press articles to link the academic discourse with the public discourse that draws on *anecdotal evidence* and often focuses more on practical solutions. Our procedure in phases 2 to 6 according to Table 1 is visualized in more detail in a flow chart (see Figure 1). The flow chart illustrates how our selection of literature was grouped (e.g., according to direct and indirect evidence). Indirect evidence covers studies not explicitly addressing videoconference fatigue but addressing phenomena related to physical and mental effort and cognitive load.

As a conceptual analysis is an iterative process, Phase 5 (integrating components) and Phase 6 (re-grouping dimensions) were repeated several times until the research team agreed on the structure of the final 4D model with its sub-dimensions. Throughout the iterations the labels for the dimensions as well as the labels and numbers of sub-dimensions were changed in search for the most comprehensive and concise solution.

During Phase 7, we presented and discussed the new 4D model of VC fatigue within the academic setting through five different project workshops and colloquia. This phase involved 29 external experts, representing a broad range of relevant disciplinary backgrounds, in the further refinement of the proposed model. The authors’ suggestions regarding the organization and theoretical elaboration of the causal factors were partly supported and partly questioned and subsequently revised again. We repeatedly discussed issues of “Face-to-Face meeting fatigue” in contrast to “Videoconference fatigue” to scrutinize both the model and the concept of videoconference fatigue. Phase 8 (see Table 1) is ongoing as we—and possibly other researchers as well—will be using the conceptual model proposed in this article to design and conduct empirical studies on VC fatigue in the future. Each phase of the research process lasted several weeks and involved individual work by all co-authors as well as regular Zoom meetings among them.

## 3. Results

**Figure 2:**
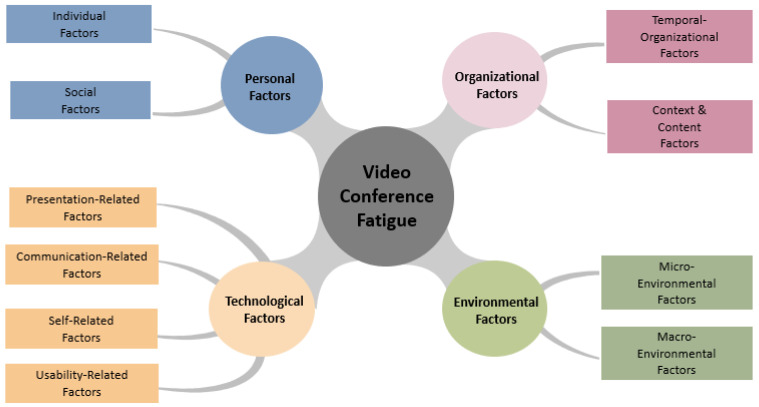
The 4-Dimensional (4D) Model of Videoconference Fatigue and its Causes.

The newly developed 4D conceptual model of videoconference fatigue entails the following key causal dimensions: (1) personal factors, (2) organizational factors, (3) technological factors and (4) environmental factors (see Figure 2). In the following sections, each dimension will be presented separately with its sub-dimensions.

### 3.1. Personal Factors

The personal factors dimension covers person-related factors that causally influence the experience of videoconference fatigue. The umbrella theory behind this dimension is the *Differential Susceptibility of Media Effects Model* (DSMM) [28], a well-established general model of media effects, that—among other things—underlines the fact that media effects differ significantly depending on people’s individual characteristics and the social context. Hence, the personal factors dimension has two sub-dimensions: individual and social factors (see Table 2).

**Table 2:** Evidence table for personal factors of videoconference fatigue.

Dimension	Sub-Dimension	Variable	Theory	Empirical Evidence		Media Coverage
				Direct	Indirect	
Personal Factors	Individual Factors	General Individual Factors	DSMM ^1^, Valkenburg and Peter [28]	Fauville et al. [15]; Queiroz et al. [29]; Oducado et al. [30]	Bennett et al. [31]	Fosslien and West Duffy [4]; Kavanagh et al. [32]
		Specific Individual Factors	DSMM ^1^, Valkenburg and Peter [28] Technostress Model; Brod [33]	Nesher Shoshan and Wehrt [34]	Peper et al. [35]	Kavanagh et al. [32]
	Social Factors	Distal Social Factors	DSMM ^1^, Valkenburg and Peter [28] UTAUT ^2^, Venkatesh et al. [36]			
		Proximal Social Factors	DSMM ^1^, Valkenburg and Peter [28] ART ^3^, Kaplan [37]	Bennett et al. [38]	Peper et al. [35]	Fosslien and West Duffy [4]; Kavanagh et al. [32]

^1^. DSMM: Differential Susceptibility of Media Effects Model. ^2^. UTAUT: Unified Theory of Acceptance and Use of Technology. ^3^. ART: Attention Restauration Theory.

#### 3.1.1. Individual Factors

*General Individual Factors:* In line with the DSMM it is assumed that VC fatigue as a media effect depends on general individual dispositions that are known to influence many types of media effects, particularly sociodemographic variables (e.g., gender, age, race, ethnicity), personality types and cognitive traits [28]. One of the first larger surveys on videoconference fatigue and its follow-up work confirmed this assumption and showed that socio-demographic variables and personality traits are linked with the intensity of experienced fatigue in relation to videoconferences [15,29,30]: Results such as that female, younger and more introverted participants suffer more from Zoom fatigue have been widely picked up by the press [39]. Cognitive factors have remained under-researched, though.

*VC-Specific Individual Factors*: It seems very plausible that the general tendency of a person to be easily fatigued and not cope well with stress and technology demands will also lead to a higher susceptibility of VC fatigue. Hence, apart from aforementioned general dispositions, there are VC-specific individual factors such as mental and physical health and fitness, stress management skills and VC skills that influence the individual’s susceptibility for experiencing exhaustion and fatigue in relation to VC. Qualitative data indicate that negative versus positive attitudes towards VC influence susceptibility to VC fatigue [34]. Further, the survey study by Oducado et al. [30] found that negative attitudes towards VC go hand in hand with higher reported levels of fatigue. Media articles on Zoom fatigue provide tips and tricks on how to better cope with the stresses and strains of frequent video conferences, and how to strengthen one’s overall physical and mental health to avoid mental and physical exhaustion [4,32]. Some researchers also suggest that educational institutions implement more health and well-being initiatives (e.g., meditation and mindfulness webinars) to foster resilience to Zoom fatigue in educators [40] (pp. 61–68).

#### 3.1.2. Social Factors

*Distal Social Factors:* Personal factors operate not only on the individual but also on the social level through the interactions between different people. The DSMM underlines how people surrounding the media user (e.g., family members, peers, colleagues) influence their attitudes towards and the use or non-use of certain media [28]. This idea is also a central part of the *Unified Theory of Acceptance and Use of Technology* (UTAUT) [36], which is one of the most-used theories in the field of technology use. UTAUT predicts that attitudes and norms in the social network of a person determine their attitudes and media use behaviors. Direct evidence is not available yet, but it can be assumed that a person with a VC-aversive social network might have more negative VC expectations. These factors are labeled distal social factors because they are *operating in the background or from a distance*, and are not directly related to an ongoing VC meeting. Media articles refer to those influences when they mention how, particularly in times of the COVID-19 pandemic, there is a lot of push towards the use of VC because institutional regulations and peer pressures demand online meetings. This could make some people adapt to the situation, while others might experience resistance and, hence, increased stress and exhaustion.

*Proximal Social Factors:* The proximal social factors are those influences of other persons that operate not in the background but in the *foreground of an ongoing videoconference*. Depending on how many and which types of people are participating in the VC, and how harmonious or conflict-laden the live interactions and collaborations go, the user might feel more or less fatigued during the process and afterwards. Prior work from the fields of meeting science and virtual collaboration and teamwork is particularly relevant in the context of proximal social factors and entitativity (i.e., the “group-ness” of a meeting [41]). This research points to *session management*, *communication norms* and *participant roles* as relevant variables that can increase or decrease the burden of meetings and, hence, increase or decrease fatigue.

While measures and practices targeting effective VC session management such as choosing a facilitator and defining clear norms and roles [42] (pp. 680–706) may not have a direct impact on VC fatigue, based on the literature it can be argued that they indirectly may play an important role. Numerous studies have shown that badly managed offline meetings have a negative impact on employees’ motivation, engagement, and energy [43,44,45]. This effect might be amplified by online meetings with ineffective social rules and regulations. An empirical study conducted by Bennett et al. [38] refers to Kaplan’s [37] *Attention Restauration Theory* (ART). ART explains mechanisms of human energy depletion and repletion that is applicable to VC, where in particular the need for sustained attention is assumed to play a crucial and energy-draining role. The establishment of specific group communication norms influences this process: First of all, having clear communication norms (e.g., using the raise hand function before speaking) is likely to result in clearer expectations, reducing the required attentional resources. Secondly, the establishment of explicit norms (e.g., to switch on the camera) may increase the feeling of connectedness and belongingness among session participants, which in turn is associated with lower levels of fatigue because the experience of social belongingness provides fresh energy [38].

In both ill- and well-managed videoconference sessions, participants have different social roles that are linked with more or less stress and exhaustion. For instance, the facilitator in a business meeting most likely has a more active and exhausting role than many of the participants. Peper et al. [35] discuss this issue in the context of educational Zoom conferences and with a focus on strategies to avoid VC fatigue. More specifically, they point to the attentional challenges that many students are facing when attending an online lecture. They also refer to challenges with nonresponsive and passive behaviors of students reported by fatigued teachers. However, such social-role related causes of videoconference fatigue can be prevented. In the context of online teaching and learning, for example, students could be guided to take more constructive roles in the virtual classroom to fight both their own and their teachers’ fatigue [35]. Last, but not least it should be noted that depending on the type of VC session, the type of activity and participant role change dramatically. Therefore, certain types of VC sessions may actually help to prevent fatigue, e.g., online yoga classes (demanding physical activity and providing the social role of a spiritual practitioner) or hanging out with friends via VC, enabling a positive feeling of togetherness through intimate and rewarding social roles [46].

### 3.2. Organizational Factors

To map potential fatiguing effects related to when, how and why videoconference technology is used, the second dimension of the model comprises organizational factors. This dimension includes both temporal–organizational factors as well as context and content factors of VC sessions that can lead to videoconference fatigue (see Table 3).

**Table 3:** Evidence table for organizational factors of videoconference fatigue.

Dimension	Sub-Dimension	Variable	Theory	Empirical Evidence		Media Coverage
				Direct	Indirect	
Organizational Factors	Temporal–Organizational Factors	Number and Duration of VC Sessions; Timing of VC Sessions	ART ^1^, Kaplan [37]	Fauville et al. [15]; Bennett et al. [38]; Karl et al. [47]; Queiroz et al. [29]; Oducado et al. [30]	Bennett et al. [31]	Fosslien and Duffy [4]; Kavanagh et al. [32]; Parker [48]
	Context and Content Factors	Anticipated Outcome of VC			Standaert et al. [49]; Garro-Abarca et al. [50]	Lufkin [51]; Sklar [52]
		Activity During VC	ART ^1^, Kaplan [37]	Queiroz et al. [29]	Cao et al. [53]	Fosslien and Duffy [4]; Kavanagh et al. [32]

^1^. ART: Attention Restauration Theory.

#### 3.2.1. Temporal–Organizational Factors

*Number and Duration of VC Sessions:* Several studies have reported that the increased number (and in some cases also increased length) of VC meetings can be linked to VC fatigue [15,29,30,47,54]. The study presented in [34], however, suggested that the *perceived* duration of a meeting may be a more relevant factor than the meeting duration per se, thus indicating the need for follow-up studies. Further, earlier work in the field of *meeting science* has shown that the number of meetings and meeting load have a negative impact on employee well-being and are associated with higher daily fatigue and subjective workload [45,55,56]. In addition, a recent large-scale analysis of multitasking behavior during remote meetings using VC indicated that the increase in number of meetings has also triggered an increase in multitasking behavior to catch up with certain tasks, which in turn can lead to reduced attention and mental fatigue [53].

*Timing of VC Sessions:* Furthermore, recent empirical work from Bennett et al. [38] looked into the nature and temporal dynamics of VC fatigue to better understand how the time of the day and more generally, the scheduling of VC sessions may play a role. The authors again draw on ART [37] and their empirical findings point to the temporal distinctness of videoconference fatigue (compared to other forms of fatigue), and underline the importance of time as a key factor influencing VC fatigue [38]. Based on analyses comparing “normal daily fatigue trajectories” with altered levels of fatigue triggered by VC events, they found that VC sessions later in the day are more likely to trigger fatigue, while mid-day VC sessions are associated with lower degrees of VC fatigue [38]. Earlier work from Bennett et al. [31] has looked into the replenishing nature of so-called “micro-breaks” (e.g., short activities that take less than 10 min and are not work-related) and found that these can be beneficial to restore psychological resources (e.g., attention). Even though the relevance of taking such micro-breaks during VC sessions has not been empirically proven yet, it is frequently mentioned as a strategy to avoid VC fatigue both in academic papers [12,38,57] and newspaper articles [4,32,48]. Similarly, several of the factors addressed above have been mentioned in articles addressing the phenomenon of VC fatigue and how to prevent it [11,12,35,57,58]. However, in most cases, their ascribed importance is not based on (strong) empirical evidence.

#### 3.2.2. Context and Content Factors

The content of a VC session comprises the variables anticipated outcome of and activity during the VC session, which are also closely linked to the proximal social factors described in Section 3.1.2 and some technology factors explained in Section 3.3.2.

*Anticipated Outcome of VC:* In VC sessions that are work- or study-related, the goal and anticipated outcome may be more explicit than in VC sessions in the private sphere or linked to leisure activities. In a business context, Standaert et al. [59,60] distinguish between four different categories of intended outcomes, i.e., exchanging information, making decisions, communicating sentiments and building relations. Based on an overview of existing literature, they argue that videoconference as a medium is less effective for the latter category. They emphasize that the desired outcome needs to be considered when deciding on an appropriate virtual meeting mode, so that ineffective and bad meetings having a negative impact on day-to-day employee well-being [43] can be avoided [59,60]. Similarly, Cichomska et al. [61] (pp. 663–679) emphasize the importance of congruence between the type of meeting, the intended outcome and the goals of individual attendees in the context of virtual meetings. Lufkin [51] therefore argues for a “mix-and-match”-approach in this respect, depending on the goal(s). For VC sessions in a leisure/private context, the underlying goals and intentions may be more latent and motivated by a desire to fulfill fundamental psychological human needs, such as the need for relatedness and belongingness [52], influence and popularity, pleasure and stimulation. These have been found to be a source of positive experiences, rather than of fatigue or other negative outcomes, when they are fulfilled [62].

*Activity during VC:* The participant’s activity during the VC session is expected to indirectly influence whether or not fatigue may occur. In work- or study-related sessions, the activity is more likely to be related to one or more tasks to be completed. In this respect, in particular the relation between the communication effectiveness and team performance—which may also strongly depend on several *proximal social factors*, e.g., participants’ roles, absence or presence of a facilitator—and the task features are of importance. The latter include e.g., the task complexity, variety, analyzability, interdependence, but also the *task–technology fit* [50,63,64]. In this respect, the study of Queiroz et al. [29] found that the use of videoconferencing for study-related tasks was associated with higher reported fatigue levels than for work-related activities.

The task can also play an important role for the level of cohesion within a group: the latter is likely to be larger when the different group members consider the task to be intrinsically rewarding [50]. Additionally, intrinsic interest in the content of a VC session provides fresh energy [38] while disengagement with a task is associated with increased mental fatigue [65]. Further, as already pointed out, multitasking behavior as part of the activity may also lead to decreased attention and increased mental fatigue [53].

### 3.3. Technological Factors

It is obvious that the experience of videoconference fatigue is determined by the technology of the respective VC system. This dimension of the VC fatigue model includes four sub-dimensions: presentation-related, communication-related, self-related and usability-related technology factors (see Table 4). Note that a more comprehensive version of this chapter is available online as an annex to this article, see Raake et al. [66].

#### 3.3.1. Presentation-Related Factors

The first sub-dimension, *presentation-related factors,* comprises all technological factors that characterize the one-way capture, processing and transmission of audio, video and audiovisual information. Technological characteristics of VC can significantly increase the visual, auditory and information integration effort and subsequent perception-related fatigue that contribute to overall VC fatigue.

*Visual Fatigue:* VC does not enable the same natural ease of *visual scene analysis* in comparison to face-to-face situations. In terms of technical factors, scene analysis of on-screen information may be affected by the chosen camera, lighting conditions, video resolution and coding, size of the participant’s video window on the screen, viewing distance and possible background effects such as virtual backgrounds or blurring. In spite of the plausibility, the authors did not find direct scientific evidence for an effect of visual scene-analysis on fatigue. However, visual fatigue because of visual display usage has been reported in various studies [67,68,69,70]. As vergence rather than accommodation seems to drive fatigue, among other factors [71], the effect depends on the actual viewing distance [67,69]. The literature indicates that any prolonged work at a computer monitor causes some level of visual fatigue, VC usage does not make a difference. It is relevant to note that typical VC display viewing distances of around 30 to 80 cm are much closer than typical social distances in face-to-face situations of above 1.2 m [72].

*Auditory Fatigue:* Multiple audio-related technological factors affect *listening effort* and hence, may cause auditory fatigue. Those factors are the employed audio signal level (volume), presence of background noise at the far or the near end, room reverberation in the own or other parties’ physical environment, the audio quality related with the microphones and or loudspeakers or headphones used, degradations due to coding and transmission impairments such as interruptions, or audio signal clipping due to echo cancellers (for an overview see [73]). A link between audio quality, intelligibility and listening effort can be established following [74] (pp. 227–267), [75]. A series of studies furthermore indicate that *low-quality audio* as well as *low-quality audiovisual stimuli* lead to fatigue [76,77,78]. *Lack of spatial audio* in VC can also increase listening effort and hence likely cause fatigue (indirect evidence) [79]. Here, further plausible causes for increased listening effort are the reduced ability of auditory stream segregation [80] in non-spatial-audio VCs and thus of solving the auditory *Cocktail Party Problem* [81], as well as a reduced efficiency in memory usage [82,83,84].

*Audiovisual Fatigue:* Information integration in humans works seamlessly under normal, every-day situations. In a VC meeting, audio and video, however, may be out of sync [85] (pp. 229–270). While the human perceptual system can adjust to asynchrony [86], studies point to a slight increase in *cognitive load* [87,88], without direct evidence for increased fatigue. The online survey conducted by Bonanomi et al. [7] using their self-developed “Online Fatigue Scale” revealed high loadings for the scale item “I felt like I had to focus twice more to really understand what was going on”, which can be related to audiovisual fatigue or communication-related factors.

#### 3.3.2. Communication-Related Factors

The main purpose of VC is interpersonal communication or interaction. Technical factors of the VC setting, however, can complicate the interpersonal exchange and, hence, produce stress and fatigue.

*Problems with Nonverbal Cues:* In VC, both the perception and production of non-verbal cues such as eye contact [14,89], vocal backchannel signals or facial expressions and gestures [89,90,91], indicating agreement and affection [92] can be hindered due to audio or video properties such as small windows on the screen. Increased efforts to produce and perceive those cues most likely lead to fatigue [14,15,46,93].

*Problems with Turn Taking*: The same holds true for turn taking. Technology-induced problems with turn taking [94] (pp. 7–55), such as lacking visibility or audibility, or delayed transmission of cues, may produce misunderstandings and thus stress, likely fostering fatigue [14,95].

*Problems with Social Bonding and Impression Formation*: Technical issues with compressed audio and video, disruptions of the connection and/or delays in the audio-visual transmission disturb the interpersonal communication on all levels including social bonding and impression formation [95,96,97,98]. Technically disrupted and delayed communication can create misunderstandings and unfavorable impressions [95,96,99] which increase cognitive load [100] and may feed into the experience of *technostress* [101] and VC fatigue. Task switching due to repair activities (e.g., trying to re-connect to the VC system, changing of window size or camera angle) create further mental load [102,103] that can contribute to fatigue, can also negatively impact the person’s impression. A further presentation-related effect relates to Hall’s theory of proxemics and social distance [14,72]. Depending on camera field-of-view, distances from camera and screen, the depicted face on screen may appear intimidatingly large. While Fauville et al. [15] seem to provide some support for this effect, the practical relevance for average set-ups and on-screen window-sizes may be negligible.

#### 3.3.3. Self-Related Factors

*Problems with Being on Camera:* Based on a text mining analysis of about three million Twitter tweets from the beginning of the COVID-19 crisis, Hacker et al. [46] derived a set of affordances and five constraints of VCs, three of which are self-related factors: *Fear of being on camera*, stress of having to be “always on”, and exposing one’s private living space [46,104,105,106]. Other authors point to *mirror anxiety* (e.g., the stress to be confronted with one’s own mirror image during the whole VC session and corresponding control of the own image) and its contribution to VC fatigue [14,15,16,91,107]. Note that some later research seems to challenge the importance of the effect, indicating improved satisfaction with media self-presentation comparing online to offline [108] and no effect on learning performance [104]. A further factor was coined as “physically trapped” [14], reflecting the restricted range of motion allowed by the camera used, and with initial evidence in Fauville et al. [15].

*Problems with Producing Communication Signals:* In a VC interaction, people also feel the need to make more effort in producing audiovisual communication signals. The technological mediation of the communication pushes participants to speak louder (e.g., with increased *vocal effort* [109,110]) and to use more intensive gestures and facial expressions (e.g., more smiles) in order to be better understood [109]. Those extra-efforts contribute to fatigue, with indirect evidence derived from Kristiansen et al. [111].

#### 3.3.4. Usability-Related Factors

Last, but not least, videoconferencing implies that all participants must deal with the respective VC system. Handling a VC system’s diverse technological features can create *technostress* [101] that might lead to *techno-exhaustion* [112] as one aspect of videoconference fatigue. According to people’s complaints on Twitter they struggle with VC technology because they miss relevant features and feel a lack of security [46]. Such concerns might trigger cognitive load and an emotional burden leading to fatigue. The degree to which operating a VC system is perceived as easy and convenient or stressful and fatiguing depends on the user’s characteristics such as their VC literacy (see Section 3.1) and on the *system’s usability* and ease of use [113,114,115].

**Table 4:** Evidence table for technological factors of videoconference fatigue.

Dimension	Sub-Dimension	Variable	Theory	Empirical Evidence		Media Coverage
				Direct	Indirect	
Technological Factors	Presentation-Related Factors	Visual Fatigue	MRT ^1^, Daft and Lengel [116]; Dennis and Kinney [117]; DPT ^2^, Evans and Stanovich [118]; Kahneman [119]; Ferran and Watts [100]		Jaschinski-Kruza [67]; Tyrrell and Leibowitz [68]; Gur et al. [69]; Kim et al. [70]	Fosslien and West Duffy [4]; Johnson [120]; Kavanagh et al. [32]
		Auditory Fatigue	MRT ^1^, Daft and Lengel [116]; Dennis and Kinney [117]; DPT ^2^, Evans and Stanovich [118]; Kahneman [119]; Ferran and Watts [100]; Auditory stream analysis; Bregman [80]	Antons [76] Arndt [77]	Raake et al. [74]; Krueger et al. [75]; Fintor et al. [79]; Baldis [82]; Deng [83]; Skowronek and Raake [84]	Johnson [120]; Kavanagh et al. [32]
		Audiovisual Fatigue	Cocktail Party Effect; Bronkhorst [81]; Proxemics: Hall [72]	Arndt [78]	Buchan [87], Eg et al. [88], Bonanomi et al. [7]	
	Communication-Related Factors	Problems with Nonverbal Cues	MRT ^1^, Daft and Lengel [116]; Dennis and Kinney [117]	Fauville et al. [15]	Abdullah et al. [93]; Hacker et al. [46]; Knapp et al. [89]; Kleinke [92]; O’Conaill [90], Riva et al. [91]	Jiang [5]; Kavanagh et al. [32]; Lee [121]
		Problems with Turn-Taking	MRT ^1^, Daft and Lengel [116]; Dennis and Kinney [117]; DPT ^2^, Evans and Stanovich [118]; Kahneman [119]; Ferran and Watts [100]		Schoenenberg [95]	Johnsonm [120]
		Problems with Social Bonding and Impression Formation	MRT ^1^, Daft and Lengel [116]; Dennis and Kinney [117]; DPT ^2^, Evans and Stanovich [118]; Kahneman [119]; Ferran and Watts [100]	Fauville et al. [15]	Powers et al. [96]; Roberts and Francis [97]; Schoenenberg et al. [95,99] Siegert and Niebuhr [98]; Ferran and Watts [100] Liefooghe et al. [102]; Rubinstein et al. [103]	Jiang [5], Johnson [120]; Kavanagh et al. [32]; Lee [121]; Montañez [122]; Robert [123]
	Self-Related Factors	Problems With Being on Camera		Fauville et al. [15]	Hacker et al. [46]; Austin et al. [104]; Bedenlier et al. [105]; Shockley et al. [106]	Jiang [5]; Kavanagh et al. [32]; Robert [123]
		Problems with producing communication signals			Croes et al. [109], Tracy et al. [110], Kristiansen et al. [111]	Brower [124]
	Usability-Related Factors	Technostress, Techno-exhaustion	Tarafdar [101]; Weinert [112]		Hacker et al. [46]; Ayyagari et al. [113]; Galluch et al. [114]; Pirkkalainen et al. [115]	Brower [124]; Fosslien and West Duffy [4]; Robert [123]

^1^. MRT: Media Richness Theory. ^2^. DPT: Dual-Process Theory.

### 3.4. Environmental Factors

The discussion of the phenomenon of videoconference fatigue came up in an exceptional time. It started during a pandemic in which most people were obliged to work and study from home. In the debate on Zoom fatigue, this dramatic change of the context in which videoconferencing takes place has hardly been considered. For this reason, the fourth dimension of the 4D-model of VC fatigue captures factors related to the environment of VCs including two sub-dimensions: micro-environmental and macro-environmental factors (see Table 5).

#### 3.4.1. Micro-Environmental Factors

The micro-environment sub-dimension comprises the variables physical and psychological micro-environment.

*Physical Micro-Environmental Factors:* The physical micro-environment includes the setting of a person’s work or study or leisure place and its context. Having to work or study or socialize from home may imply using different equipment and being in other contextual conditions than usually. A recent review on the impact of the interior office environment on well-being identified a negative relationship of background noise on fatigue or alertness [125]. For climate control, the relationships investigated were not obvious, while plants were shown to have a positive association to fatigue reduction. Even though no study investigated the impact of such direct contextual factors on VC fatigue so far, the direct physical environment may, at least to a small extent, influence how fatiguing VC meetings are perceived to be.

*Psychological Micro-Environmental Factors:* Psychological micro-environmental aspects are expected to affect fatigue. Recent media articles addressed psychological environmental factors such as issues with requests and distraction associated to different social roles [4,124]. According to the *work–home resources model* by Ten Brummelhuis and Bakker [126], work and home reciprocally affect each other in a positive and negative way. Particularly, simultaneous presence of time-sensitive demands from different domains, e.g., work and home, and role conflicts may occur and cause distraction, an elevated level of stress and, as a consequence, fatigue. For example, having to organize home schooling or lunch for the kids and participate in work-related meetings at the same time will lead to a conflict of demands and roles. Such conflicts, also called *work–home interference*, were found to be causally related to later exhaustion and work pressure in a study by Demerouti et al. [127]. Furthermore, a clear definition of work and leisure or family time often disappears in a work-from-home setting. The resulting flexibility may be beneficial: As Dettmers et al. [128] have shown for parents, morning demands predicted morning fatigue after arrival at work, however flexible working hours buffered the effect. Even though this study did not investigate work from home explicitly, it provided first insights into positive effects of flexible scheduling. On the other hand, a study among academic staff reported fatigue and stress to be increased for people who telecommunicated more often, implying that telecommunication was necessary during home-office time [129] (pp. 308–312). Telecommunication from home during the pandemic was associated to work-related fatigue and to more conflicts of the domains home and work [130]. It seems reasonable to assume a medium impact of psychological environmental factors on videoconferencing fatigue.

#### 3.4.2. Macro-Environmental Factors

The macro-environment sub-dimension represents the overall situation of a person in the context of the society and entails both needs and opportunities.

*Macro-Environmental Needs*: The model of human motivation by Maslow [131] represents a theoretical basis for human needs (BNT: *Basic Needs Theory*). Besides physiological needs, Maslow [131] describes the need for safety and stability on the second level of his model, emphasizing its importance for human well-being. Media highlighted how people may have experienced a loss of control due to the pandemic situation [124]. Health has been threatened directly and sudden actions to oppose the pandemic were pulling people out of their common routines, possibly causing higher levels of insecurity and unpredictability. Certainly, individual coping strategies (see Section 3.1: personal factors) influence the subjective assessment of current security and stability. However, no study has investigated the effects of loss of security and stability on videoconferencing fatigue so far. Nevertheless, several studies showed how global mental health, especially symptoms of depression and anxiety, got worse during the pandemic [132,133,134,135,136,137,138,139]. Depressiveness often includes tiredness and a lack of energy [140]. As a consequence, people who feel more depressed may also experience videoconferencing as more exhausting and fatiguing. Due to the complex interaction of different variables, the impact of macro-environmental needs on videoconferencing fatigue can vary strongly between individuals but is expected to be at least small.

*Macro-Environmental Opportunities:* Another reason for impaired health in times of pandemic could be the lack of macro-environmental opportunities. In Maslow’s [131] model, these aspects are represented by the need for love, self-esteem and self-actualization. Besides social relationships, the possibility to be physically active and gain diverse physiological inputs may be important to not easily feel fatigued.

The media addressed these topics by describing how people are moving less [124], are avoiding public or family get-togethers [141], and by giving tips to prevent COVID-19 related feelings of fatigue [122,123,142]. Research partly supported these presumptions, finding higher levels of daily-life fatigue in females and people who were living alone during the crisis [143]. A review article by Kniffin et al. [144] explained the detrimental effect of pandemic-related changes in working conditions and of social distancing based on knowledge from work and organizational psychology. They described how the situation contributed to, for example, loneliness and rumination, leading to a chronic stress and exhaustion. In sum, the impact of macro-environmental opportunities on videoconferencing fatigue is highly related to other (sub)dimensions. Nevertheless, this factor is expected to have at least a small effect on videoconferencing fatigue.

**Table 5:** Evidence table for environmental factors of videoconference fatigue.

Dimension	Sub-Dimension	Variable	Theory	Empirical Evidence		Media Coverage
				Direct	Indirect	
Environmental Factors	Micro-Environmental Factors	Physical Micro Environment				
		Psychological Micro Environment	WHRM ^1^, ten Brummelhuis and Bakker [126]		Palumbo [130]	Brower [124]; Fosslien and West Duffy [4]
	Macro-Environmental Factors	Macro Environmental Needs	BNT ^2^, Maslow [131]			Brower [124]; Montañez [122]
		Macro Environmental Opportunities	BNT ^2^, Maslow [131]			Ahmed [142]; Meichtry, Suyden and Barnett [141]; Brower [124]; Montañez [122]; Robert [123]

^1^. WHRM: Work–Home Resources Model. ^2^. BNT: Basic Needs Theory.

## 4. Discussion

Previous theoretical conceptualizations of Zoom fatigue have focused a lot on the technology mediated communication in an ongoing VC session [10,14]. Comparing VC sessions with face-to-face meetings and drawing on *Media Naturalness Theory* [145], their main idea is that the unnatural technology mediated situation creates additional sensory and cognitive load and, hence, fatigue. While we agree and regard technological factors at the root of Zoom fatigue, our 4D model also stresses the relevance of personal, organizational, and environmental factors often neglected in both public and academic discourses.

According to our comprehensive *4D model of videoconference fatigue*, the phenomenon can be explained by (1) personal, (2) organizational, (3) technological and (4) environmental factors. While face-to-face meetings might be exhausting as well, anecdotal evidence discussed in the media and empirical data presented in academic papers point to the fact that social interaction via a videoconference system is often particularly stressful and fatiguing. However, countermeasures to fight increased exhaustion related to VC are available.

Our 4D model states that *personal factors* play a crucial role: People with good mental and physical health and the necessary VC skills should experience less VC fatigue. Hence, improving these factors should reduce a person’s susceptibility to Zoom fatigue. Furthermore, good videoconference session management, shared communication norms and positive interpersonal relations among participants of a VC session can be a buffer against exhaustion, because the positive social experience re-energizes VC participants.

Regarding *organizational factors* there seems to be a fairly broad consensus between public and academic debates: What makes VC sessions particularly exhausting is their number, duration, timing, and burstiness. Scheduling VC sessions wisely, keeping them short and allowing for enough breaks in-between eliminates main causes of VC fatigue. Furthermore, to keep the energy level of participants up, it is important to involve everyone actively in the interaction, to try to trigger participants’ intrinsic interest and avoid energy-draining multi-tasking. Being aware of the degrees of freedom in managing VC sessions and of the benefits of organizing everything wisely is a prerequisite of fruitful VC use.

*Technological factors* are at the core of videoconference fatigue. In fact, the technological mediation of interpersonal communication brings about a lot of stresses and strains. Research has demonstrated that participating in a VC session requires increased visual, auditory and vocal efforts as well as cognitive load that all create exhaustion. Some of these problems could be eliminated or at least diminished with better internet connectivity, improved usability of VC systems, more appropriate technical equipment (e.g., wide-angle camera, high end microphone and loudspeaker) and innovative technology such as spatial audio and augmented reality features.

Last not least, our 4D-model of videoconference fatigue with *environmental factors* as its fourth dimension reminds everyone that VC sessions do not take place in a vacuum but in the micro-environment of the participant’s workplace or home. Particularly in a working-from-home situation, VC fatigue can be caused by conflicting roles and demands of the work and home context. One way to fight VC fatigue could be to enhance coping with work–home interferences. Finally, it should not be forgotten that the macro environment also shapes VC experiences and outcomes. In times of the world-wide COVID-19 pandemic it is almost impossible to disentangle the exhaustion caused by pandemic-related disruptions of private and professional lives and that caused by inconvenient VC sessions.

The 4D-model of videoconference fatigue is meant to inspire *future research*. One fruitful research approach to establish which factors of the model are main causes or rather minor causes of VC fatigue would be a well-designed series of experiments. Experimental assessment would also be helpful to evaluate which counter-measures against VC fatigue are most effective in which contexts. Furthermore, we need to collect more information about people’s pre-, peri- and post-pandemic experiences with videoconference fatigue, e.g., via interview studies, focus group discussions, surveys or social media data analyses. Longitudinal studies could be particularly helpful to investigate how people’s expectations, skills, behaviors and outcomes of videoconferencing change over time. Complementing subjective data from interviews and questionnaires, the integration of neurophysiological measurements of fatigue would enhance the field [10].

## 5. Limitations

Despite its universal approach, the presented conceptual analysis of videoconference fatigue has its limitations. The analysis has been conducted by an interdisciplinary research team with experts from psychology, communication science and engineering. It was validated by discussions with further colleagues from these three fields and related social sciences (e.g., sociology). The conceptual analysis methodology involved searching for and analyzing a large body of interdisciplinary literature that is presented both in text and tables. However, it has not been the goal of this paper to provide a systematic literature review. Similarly, the goal was not to provide an exhaustive overview of relevant theories. In the proposed model and based on the analytic activities and external expert involvement in phases 2 and 7, only the most prominent theories that were identified, were included. However, future work may lead to the integration of additional relevant theories, based on new empirical insights and literature on VC fatigue.

As our literature search was mostly limited to English-language academic and media sources and our research group is based in central Europe, we need to admit that our perspective might have a bias toward the Western world and the Global North. Furthermore, due to length limitations, we were not able to further elaborate the multiple interrelations between the dimensions, sub-dimensions and mediating effects in this paper. Lastly, we wish to remind readers that empirical (and particularly experimental) Zoom fatigue research is at an early stage. Hence, the included empirical studies directly addressing VC fatigue are relatively small in number, often of limited methodological rigor and sometimes provide contradicting evidence or null-findings. While it sometimes was possible to argue with reason which sub-factors might be more influential than others, future experimental studies may help to clarify causes of Zoom fatigue.

## 6. Conclusions

Videoconference fatigue is a negative health outcome of VC use that should be taken seriously. To avoid short-term fatigue and long-term exhaustion or burn-out, it is important to understand the main factors that contribute to VC fatigue. The new 4D-model of VC fatigue helps to identify and structure relevant personal, organizational, technological, and environmental factors and to plan future studies in a structured way. Research on VC fatigue can go hand in hand with the identification and evaluation of counter-measures to fight VC fatigue. Focusing on issues of fatigue related to videoconference meetings does not intend to idealize face-to-face meetings. We encourage others to advance knowledge on videoconference fatigue by also broadening our understanding of fatigue in relation to face-to-face meetings, also considering new VC technology such as eXtended Reality (XR). Furthermore, VC fatigue needs to be weighed against several advantages of VC, particularly in situations when VC sessions relief us from daily stresses and strains (e.g., avoiding commute stress and rush-hour stress when working from home). Most likely, we are heading to a hybrid future with daily face-to-face and videoconference meetings that both should spare us from fatigue but save or even fuel our physical and mental energy.

## Figures and Tables

**Table 1:** Eight phases of the conceptual analysis (revised version of the approach used by Bröder et al. [22]).

Main Steps	Analytic Activities
Phase 1	Selecting the target concept for analysis, searching for and mapping relevant data sources
Phase 2	Extensively reading and categorizing the selected data
Phase 3	Identifying and naming the dimensions and components of the target concept
Phase 4	Deconstructing and categorizing the target concept’s attributes, characteristics and assumptions
Phase 5	Integrating the components of the target concept
Phase 6	Grouping, synthesizing and resynthesizing the dimensions of the target concept
Phase 7	Validating the results of the conceptual analysis of the target concept through critical discussions within the academic setting
Phase 8	Identifying hypotheses and implications for future research and development regarding the target concept

## Data Availability

Not applicable.
